# Origin of heterogeneous spiking patterns from continuously distributed ion channel densities: a computational study in spinal dorsal horn neurons

**DOI:** 10.1113/JP275240

**Published:** 2018-02-21

**Authors:** Arjun Balachandar, Steven A. Prescott

**Affiliations:** ^1^ Neurosciences and Mental Health The Hospital for Sick Children Toronto Canada; ^2^ Department of Physiology and the Institute of Biomaterials and Biomedical Engineering University of Toronto Toronto Canada

**Keywords:** spiking pattern, Spinal cord, dorsal horn, Neuronal excitability, classification, computational modeling

## Abstract

**Key points:**

- Distinct spiking patterns may arise from qualitative differences in ion channel expression (i.e. when different neurons express distinct ion channels) and/or when quantitative differences in expression levels qualitatively alter the spike generation process.
- We hypothesized that spiking patterns in neurons of the superficial dorsal horn (SDH) of spinal cord reflect both mechanisms.
- We reproduced SDH neuron spiking patterns by varying densities of K_V_1‐ and A‐type potassium conductances. Plotting the spiking patterns that emerge from different density combinations revealed spiking‐pattern regions separated by boundaries (bifurcations).
- This map suggests that certain spiking pattern combinations occur when the distribution of potassium channel densities straddle boundaries, whereas other spiking patterns reflect distinct patterns of ion channel expression. The former mechanism may explain why certain spiking patterns co‐occur in genetically identified neuron types.
- We also present algorithms to predict spiking pattern proportions from ion channel density distributions, and vice versa.

**Abstract:**

Neurons are often classified by spiking pattern. Yet, some neurons exhibit distinct patterns under subtly different test conditions, which suggests that they operate near an abrupt transition, or bifurcation. A set of such neurons may exhibit heterogeneous spiking patterns not because of qualitative differences in which ion channels they express, but rather because quantitative differences in expression levels cause neurons to operate on opposite sides of a bifurcation. Neurons in the spinal dorsal horn, for example, respond to somatic current injection with patterns that include tonic, single, gap, delayed and reluctant spiking. It is unclear whether these patterns reflect five cell populations (defined by distinct ion channel expression patterns), heterogeneity within a single population, or some combination thereof. We reproduced all five spiking patterns in a computational model by varying the densities of a low‐threshold (K_V_1‐type) potassium conductance and an inactivating (A‐type) potassium conductance and found that single, gap, delayed and reluctant spiking arise when the joint probability distribution of those channel densities spans two intersecting bifurcations that divide the parameter space into quadrants, each associated with a different spiking pattern. Tonic spiking likely arises from a separate distribution of potassium channel densities. These results argue in favour of two cell populations, one characterized by tonic spiking and the other by heterogeneous spiking patterns. We present algorithms to predict spiking pattern proportions based on ion channel density distributions and, conversely, to estimate ion channel density distributions based on spiking pattern proportions. The implications for classifying cells based on spiking pattern are discussed.

## Key points

- Distinct spiking patterns may arise from qualitative differences in ion channel expression (i.e. when different neurons express distinct ion channels) and/or when quantitative differences in expression levels qualitatively alter the spike generation process.
- We hypothesized that spiking patterns in neurons of the superficial dorsal horn (SDH) of spinal cord reflect both mechanisms.
- We reproduced SDH neuron spiking patterns by varying densities of K_V_1‐ and A‐type potassium conductances. Plotting the spiking patterns that emerge from different density combinations revealed spiking‐pattern regions separated by boundaries (bifurcations).
- This map suggests that certain spiking pattern combinations occur when the distribution of potassium channel densities straddle boundaries, whereas other spiking patterns reflect distinct patterns of ion channel expression. The former mechanism may explain why certain spiking patterns co‐occur in genetically identified neuron types.
- We also present algorithms to predict spiking pattern proportions from ion channel density distributions, and vice versa.

## Introduction

Neurons can be classified using various criteria such as their electrophysiological properties (including spiking pattern), morphology and expression of neurochemical and genetic markers. Classification schemes ideally consider combinations of factors (e.g. Cauli *et al*. 2000; Nelson *et al*. 2006; Ascoli *et al*. 2008; Hamilton *et al*. 2012; Zeng & Sanes, 2017) to identify robust clusters representing bona fide neuron ‘types’. Accurately identifying neuron types is critical for studying how developmental programmes lead to neuronal diversity and how that diversity is utilized to form complicated neural circuits. Yet certain populations of neurons seem to defy classification. A good example is neurons in the superficial dorsal horn (SDH) of the spinal cord (Graham *et al*. 2007a; Todd, 2017).

**Figure 1:**
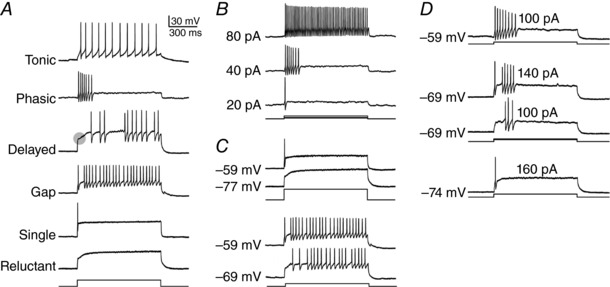
Heterogeneous spiking patterns in SDH neurons *A*, sample recordings from five SDH neurons showing the range of spiking patterns evoked by sustained somatic current injection. Note that delayed spiking is preceded by a sharp inflection (shaded circle) indicative of an A‐type potassium current; the inflection is replaced by an initial spike in gap spiking. However, spiking pattern is not a truly innate property of the neuron since it can vary with stimulus intensity (*B*) or pre‐stimulus membrane potential (*C*). *D*, example of a neuron in which different combinations of stimulus intensity and membrane potential yielded four different spiking patterns. Modified from Prescott and De Koninck (2002).

The SDH – defined as lamina I and II of spinal cord – plays an important role in the early processing of somatosensory information, especially thermal and nociceptive input (for reviews, see Ribeiro‐da‐Silva & De Koninck, 2008; Todd, 2010; Prescott & Ratté, 2012; Cordero‐Erausquin *et al*. 2016; Peirs & Seal, 2016). Only a minority (∼5%) of neurons in lamina I project to supraspinal targets (Spike *et al*. 2003). The remaining neurons, including all of those in lamina II, are local interneurons of which roughly one‐third are inhibitory and two‐thirds are excitatory (Polgar *et al*. 2003). SDH neurons exhibit diverse spiking patterns (Fig. 1 *A*). Paired recordings (Lu & Perl, 2005) and correlation with immunocytochemical markers (Yasaka *et al*. 2010) have revealed differences in the spiking patterns of excitatory and inhibitory neurons. Molecular genetic tools have dramatically accelerated this characterization (Duan *et al*. 2014; Kardon *et al*. 2014; Peirs *et al*. 2015; Petitjean *et al*. 2015; Abraira *et al*. 2016; Cheng *et al*. 2017). But genetically identified cell types can be surprisingly heterogeneous when it comes to spiking pattern (Heinke *et al*. 2004; Punnakkal *et al*. 2014; Smith *et al*. 2015). Linking gene expression patterns with electrophysiological phenotype on a cell‐by‐cell basis is now conceivable with the advent of single‐cell RNAseq (Cadwell *et al*. 2016; Fuzik *et al*. 2016; Poulin *et al*. 2016; Johnson & Walsh, 2017), but this will require more detailed understanding of electrophysiological heterogeneity.

Qualitative differences in spiking pattern are often assumed to arise from qualitative differences in ion channel expression (i.e. expression of different ion channels). But quantitative differences in ion channel expression (i.e. expression of the same ion channels but at different levels, or densities) can also yield distinct spiking patterns if they qualitatively alter spike generation dynamics, which reflect the *non‐linear* interaction between ion channels (Izhikevich, 2007; Prescott *et al*. 2008). A qualitative (discontinuous) change in output caused by continuous variation of a parameter is referred to as a bifurcation. Slight variations in ion channel density may cause a neuron to exhibit very different spiking patterns if that variation shifts the neuron across a bifurcation. Variations in stimulus intensity or pre‐stimulus membrane potential can similarly affect the spiking pattern if a bifurcation is crossed. It is, therefore, notable that spiking patterns in some SDH neurons are sensitive to stimulus intensity and/or pre‐stimulus membrane potential (Fig. 1 *B*–*D*), as this suggests that some SDH neurons do indeed operate near a bifurcation. A direct corollary of this is that a population of neurons may exhibit different spiking patterns because variation in ion channel expression across the population causes subsets of neurons to operate on opposite sides of a bifurcation. Thus, a population of neurons may exhibit heterogeneous spiking patterns because of qualitative differences in ion channel expression or because an ion channel density distribution straddles a bifurcation (where *distribution* refers to the variance in ion channel density across neurons). We hypothesized that the latter contributes to explaining the heterogeneous spiking patterns observed in SDH neurons.

To test our hypothesis, we reproduced tonic, single, gap, delayed and reluctant spiking in a simple conductance‐based computer model. Then, following the approach used by Le Franc and Le Masson (2010) to study spiking patterns in deep dorsal horn neurons, we systematically co‐varied the densities of potassium channels responsible for SDH neuron spiking patterns in order to map out parameter combinations where the model switched patterns (i.e. bifurcated). The resulting map reveals which spiking pattern combinations are likely to arise from a single distribution of ion channel densities straddling a bifurcation and which arise from separate ion channel distributions. The implications for SDH neuron classification are discussed.

## Methods

### Modified Morris–Lecar model

Simulations were conducted using a modified version of the Morris–Lecar model (Rinzel & Ermentrout, 1989; Prescott *et al*. 2008). The starting model contained only a leak conductance *g*_leak_, an instantaneously activating sodium conductance *g*_Na_ and a delayed rectifier potassium conductance *g*_K,dr_. To this model we added a low‐threshold non‐inactivating (K_v_1‐type) potassium conductance *g*_K,lt_ and an inactivating (A‐type) potassium conductance *g*_K,A_. Activation of the latter was modelled after Connor and Stevens (1971). The system is described by:

(1) CdVdt=I stim −g¯ Na m∞(V)(V−E Na )−g¯K, dr w(V−EK)−g¯K, lt z(V−EK)−g¯K,Aa4b(V−EK)−g leak (V−E leak )

(2) $$\frac{dx}{dt}=\varphi_{x}\;\frac{x_{\infty }\left(V\right)-x}{\tau_{x}\left(V\right)}$$

where *x* corresponds to the gating variables, *w*, *z*, *a* or *b*. Because *m* is assumed to activate instantaneously with changes in *V*, it is always at steady state. Steady‐state activation curves and voltage‐dependent time constants are modelled according to:

(3) $$m_{\infty }\left(V\right)=\;0.5\left[1+\tanh\left(\frac{V-\beta_{m}}{\gamma_{m}}\right)\right]$$

(4) $$w_{\infty }\left(V\right)=\;0.5\left[1+\tanh\left(\frac{V-\beta_{w}}{\gamma_{w}}\right)\right]$$

(5) $$z_{\infty }\left(V\right)=\;0.5\left[1+\tanh\left(\frac{V-\beta_{z}}{\gamma_{z}}\right)\right]$$

(6) $$\begin{array}{ccc} a_{\infty }\left(V\right) & = & \frac{1}{1+e^{-\left(\frac{V+60}{8.5}\right)}}\\ b_{\infty }\left(V\right) & = & \frac{1}{1+e^{\left(\frac{V+78}{6}\right)}} \end{array}$$

(7) τw(V)=1/ cosh V−βw2γw

(8) τz(V)=1/ cosh V−βz2γz

(9) $$\begin{array}{c} \tau_{a}\left(V\right)=\frac{1}{e^{\left(\frac{V+35.82}{19.69}\right)}+e^{-\left(\frac{V+79.69}{12.7}\right)}}\;+0.37 \end{array}$$

(10) $$\begin{array}{c} \tau_{b}\left(V\right)=\left\{\begin{array}{cc} 19, & V>-63\\ \frac{1}{e^{\left(\frac{V+46.05}{5}\right)}+e^{-\left(\frac{V+238.4}{37.45}\right)}}, & V<-63 \end{array}\right. \end{array}$$

*C* = 2 μF cm^−2^, *E*_Na_ *= *50 mV, *E*_K_ = –100 mV, *E*_leak_ *= *–70 mV, ${\bar{g}}_{\mathrm{Na}}$ = 20 mS cm^−2^, ${\bar{g}}_{K,\mathrm{dr}}$ = 20 mS cm^−2^, ${\bar{g}}_{\mathrm{leak}}$ = 2 mS cm^−2^, *ϕ_w_* = 0.15, *ϕ_z_* = 0.15, *ϕ_a_ = *1.0, *ϕ_b_ = *1.0, *β_m_* = −1.2 mV, *β_w_* = −10 mV, *β_z_* = −21 mV, *γ_m_* = 18 mV, *γ_w_* = 10 mV, *γ_z_* = 15 mV, and ${\bar{g}}_{K,\mathrm{lt}}$ and ${\bar{g}}_{K,A}$ were set to values identified in the Results, or were systematically varied in 0.1 mS cm^−2^ increments to map the parameter space giving rise to different spiking patterns. This procedure was repeated across multiple stimulus intensities (from 30 to 110 μA cm^−2^, at 5 μA cm^−2^ intervals) and different pre‐stimulus membrane potentials (see Results). Equations were numerically integrated in MATLAB (The MathWorks Inc., Natick, MA, USA) using the Euler method and a 0.1 ms time step. Each simulation was run for 250 ms to reach steady‐state before the application of a stimulating current (*I*_stim_) for 400 ms. No dendritic or axonal compartments were included in our model because, although spiking pattern and dendritic morphology are somewhat correlated, there is no evidence of a causal relationship and, furthermore, spiking pattern is defined by the response to somatic current injection (as opposed to dendritic stimulation) and those spikes originate in or near the soma.

After each simulation, neurons were classified as tonic, single, delayed, gap, or reluctant spiking based on the following criteria. Firstly, all neurons that did not spike during stimulation were labelled reluctant spiking. Neurons that produced only one spike, but did not satisfy the criteria for delayed spiking (see below) were labelled as single spiking. The remaining multi‐spike neurons were categorized based on the inter‐spike intervals (ISI) of their initial spikes. Neurons that exhibited an initial spike, but had a delay before the second spike (i.e. a ‘gap’) of greater than 1.5 times the ISI between the second and third spikes were labelled gap spiking. Those that had a delay before firing their first spike of more than 1.5 times the ISI between the first two spikes were labelled delayed spiking. Neurons that fired only one spike with a delay >100 ms were also considered delayed spiking. Neurons firing multiple spikes with neither a gap nor a delay (as defined above) were labelled tonic spiking.

### Predicting single neuron conductance densities from spiking pattern sequences

Running the model with specified values of ${\bar{g}}_{K,\mathrm{lt}}$ and ${\bar{g}}_{K,A}$ gives a spiking pattern that depends on *I*_stim_. Re‐testing different *I*_stim_ gives a sequence of spiking patterns. Working in the opposite direction, if one knows the spiking pattern evoked by a certain value of *I*_stim_, estimates of ${\bar{g}}_{K,\mathrm{lt}}$ and ${\bar{g}}_{K,A}$ are only weakly constrained (i.e. there are many different potassium channel densities that could yield a given pattern). But if one knows the sequence of spiking patterns evoked by a sequence of *I*_stim_, the estimation of ${\bar{g}}_{K,\mathrm{lt}}$ and ${\bar{g}}_{K,A}$ becomes more tightly constrained. To estimate those densities, all points (${\bar{g}}_{K,\mathrm{lt}}$,${\bar{g}}_{K,A}$) that yield the observed spiking pattern for a given value of *I*_stim_ were identified, and this was repeated for *I*_stim_ from 50 to 80 μA cm^−2^ tested at 5 μA cm^−2^ intervals. The *intersection* of those points across planes was then identified, thus revealing values of ${\bar{g}}_{K,\mathrm{lt}}$ and$\;{\bar{g}}_{K,A}$ that give the correct spiking patterns for *all I_s_*_tim_.

### Calculating spiking pattern proportions from the joint distribution of ion channel densities

For a population of neurons, ${\bar{g}}_{K,\mathrm{lt}}$ and ${\bar{g}}_{K,A}$ were assumed to have Gaussian distributions with mean values μ_K,lt_ and μ_K,A_, and standard deviations σ_K,lt_ and σ_K,A_, respectively. A correlation coefficient ρ (ranging from −1 to 1) must be included to account for any correlation between ${\bar{g}}_{K,\mathrm{lt}}$ and $\;{\bar{g}}_{K,A}$. Combining these two univariate normal distributions, and any correlation between them, gives a bivariate normal distribution (BND). The probability density function of the BND is given by:

(11) $$P\left(x,y\right)=\frac{1}{2\pi \sigma_{x}\sigma_{y}\sqrt{1-\rho^{2}}}\;e^{\left[-\frac{z}{2\left(1-\rho^{2}\right)}\right]}$$

(12) $$\begin{array}{ccc} z\; & = & \frac{{\left(x-\mu_{x}\right)}^{2}}{{\sigma_{x}}^{2}}\;-\frac{2\rho \left(x-\mu_{x}\right)\left(y-\mu_{y}\right)}{\sigma_{x}\sigma_{y}}+\frac{{\left(y-\mu_{y}\right)}^{2}}{{\sigma_{y}}^{2}} \end{array}$$

where *x* and *y* represent *g*_K,lt_ and *g*_K,A_, respectively. Equation (12) describes an elliptic paraboloid surface, where cross‐sections parallel to the *xy*‐plane are ellipses with centre at (*μ_x_, μ_y_*) = (μ_K,lt_, μ_K,A_) and rotation determined by the correlation coefficient ρ. Integrating the distribution over the region in the parameter space corresponding to a certain spiking pattern yields the volume within that region. Since the total volume under a BND is, by definition, 1, the volume over each spiking pattern region *R_i_* represents the proportion of neurons with spiking pattern *i*, where *i = *1–5 and corresponds to reluctant, single, delayed, gap and tonic spiking, respectively. Hence, the proportion *V_i_* of each firing‐pattern *i* within a given model neuron population is given by:

(13) $$V_{i}=\int \int_{R_{i}}P(x,y)dxdy\;$$

The definite double integral in eqn (13) was computed numerically using two‐dimensional trapezoidal integration.

### Estimating the joint distribution of ion channel densities from spiking pattern proportions

Working in the opposite direction from the calculations described above, we developed an algorithm to estimate the underlying ion channel distributions that best account for the proportions of different spiking patterns observed within a sample of neurons. Specifically, a geometric‐based optimization algorithm was created to find a BND describing $\bar{g}$_K,lt_ and $\bar{g}$_K,A_ such that the BND volume within each spiking pattern region reproduces an observed set of spiking pattern proportions. The algorithm was implemented as follows (where *μ_x_*_,_ *_k_* and *μ_y_*_,_ *_k_* represent the estimation in the *k*‐th iteration of μ_K,lt_ and μ_K,A_, respectively). We started with an arbitrarily chosen BND at the centre of the parameter space (i.e. for a 20 mS cm^−2^ × 20 mS cm^−2^ plot, *μ_x_*_,0_ *= *10 mS cm^−2^; *μ_y_*_,0_ = 10 mS cm^−2^), with no correlation (ρ = 0) and pre‐set *σ_x_* = σ_K,lt_ = 1 mS cm^−2^ and *σ_y_* = σ_K,A_ = 1 mS cm^−2^. Volumes under the distribution (*V*_calc_) were computed using eqn (13). The calculated proportions *V*_calc,i_ and the target proportions *V*_target,i_ were then compared for each firing‐pattern *i*, yielding a set of error terms *E*_i_ where:

(14) Ei=V target ,i−V calc ,i;i=0,…,5

(15) $$\mathrm{MaxError}\;=\max\left\{\left|E_{1}\right|,\ldots ,\left|E_{5}\right|\right\}\;$$

In words, MaxError is the highest absolute‐value difference between the calculated and target volumes for any spiking pattern. In additional tests, a finite number of samples (*n*) was randomly drawn from the BND and the target spiking pattern proportion was calculated from the number of samples falling within each spiking pattern region. This models the experimental scenario in which *V*_target,_ *_i_* are estimated from a limited sampling of neurons rather than being strictly known.

Step 1 – modifying ρ. The correlation coefficient ρ was varied systematically from −0.9 to +0.9 by increments of 0.1, thus producing a set of rotated BNDs. MaxError was calculated for each BND and was compared to arbitrarily chosen error thresholds δ = 0.001 and ε = 0.003, which correspond to 0.1% and 0.3% errors, respectively. If MaxError fell below ε, the algorithm proceeded to vary ρ in increments of 0.01 from −0.99 to +0.99. If instead MaxError was less than δ, the algorithm ended (see below). If MaxError remained above δ, the value of ρ yielding the smallest error (ρ_optimized_) was carried forward to step 2.
Step 2 – optimizing μ. The next step was to move the centre of the BND from *M*_0_ = (*μ_x_*_,0_, *μ_y_*_,0_) to a new locus *M*_1_ = (*μ_x_*_,1_, *μ_y_*_,1_). To efficiently reduce the error between calculated and target volumes, the centre of the distribution was moved towards regions where *V*_target,_ *_i_* > *V*_calc,_ *_i_* and away from those where *V*_target,_ *_i_* < *V*_calc,_ *_i_*. To determine the direction to move *M*, the centroid of each region was computed using a weighted average of all points in the region, given by:
  (16) $$C_{i}=\left(\frac{\sum_{j=1}^{n_{i}}p_{j,x}}{n_{i}},\frac{\sum_{j=1}^{n_{i}}p_{j,y}}{n_{i}}\right)\;;\;i\;=\;0,\ldots ,5$$
  where *n_i_* is the number of points in *R_i_*, *j* is any integer ranging from 1 to *n_i_*, and *p_j_* = (*p_j_*_,_ *_x_*, *p_j_*_,_ *_y_*) is the corresponding *j*‐th point in *R_i_*. Next, the direction from *M* to each point *R_i_* was determined by drawing a vector $\vec{d_{i}}$ from *M* to each corresponding centroid *C_i_* given by:
  (17) $$\;\vec{d_{i}}=\vec{C_{i}}\;-\vec{M_{0}}$$
  To calculate the effect of each region on the centre of the distribution (scaled by the amount of error), each vector $\vec{d_{i}}$ was divided by its magnitude to give a unit vector in the direction of each centroid, and was then multiplied by the respective error‐term *E*_i_ to give a scaled vector $\vec{e_{i}}$ where:
  (18) $$\;\vec{e_{i}}=E_{i}\;\frac{\vec{d_{i}}}{\left\|\vec{d_{i}}\right\|}$$
  Once each error‐scaled vector was determined, *M = M_0_* was moved to a new point *M = M*_1_ by taking the net sum of all vectors $\vec{e_{i}}$ and adding the resulting vector to *M*_0_, yielding
  (19) $$\;\vec{M_{1}}=\vec{M_{0}}\;+\sum_{i\;=\;1}^{5}\vec{e_{i}}$$
  (20) $$\Rightarrow \vec{M_{1}}=\left(\mu_{x,1},\mu_{y,1}\right)$$
  where eqn (20) describes the new centre of the BND.

Step 1 was then repeated using the new BND, and MaxError was calculated again for each value of ρ. If MaxError was still >δ for all BNDs centred at *M*_1_, the algorithm repeated Step 2. This sequence was repeated *k*‐iterations until MaxError fell below δ = 0.001 or the algorithm converged on a point *M_k_*_+1_. The algorithm returned estimates for the following parameters: μ_K,lt_ = *μ_x_*_,_ *_k_*_+1_, μ_K,A_ = *μ_y_*_,_ *_k_*_+1_, and *ρ = ρ*_optimized_. Adding a step to fit σ caused the algorithm to run very slowly; instead, the two‐step algorithm was re‐run with σ_K,lt_ and σ_K,A_ set, at 0.05 mS cm^−2^ intervals, to values between 0.4 and 1.6 mS cm^−2^. Values of σ_K,lt_ and σ_K,A_ yielding the lowest MaxError were considered the best estimates. This process thus estimated the full set of distribution parameters: μ_K,lt_, μ_K,A_, ρ_optimized_, σ_K,lt_ and σ_K,A_.

All MATLAB code is available at http://prescottlab.ca.

## Results

### Qualitative reproduction of different spiking patterns in a computational model

Our first step was to reproduce the spiking patterns observed experimentally in SDH neurons during sustained somatic current injection. Sample responses in Fig. 1 *A* each come from a different neuron, thus highlighting the diversity of spiking pattern across neurons. On the other hand, sample responses in Fig. 1 *B*–*D* highlight the sensitivity of spiking pattern within a given neuron to test conditions such as stimulus intensity and/or pre‐stimulus membrane potential. The sensitivity of spiking pattern to test conditions tends to complicate classification, but can be harnessed to strengthen classification and glean addition information if properly addressed (see below and Discussion). All sample responses to somatic current injection in Fig. 1 come from previously published recordings from rat lamina I neurons (Prescott & De Koninck, 2002).

**Figure 2:**
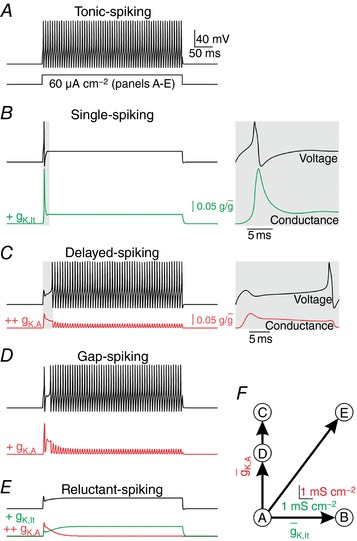
Reproduction of SDH neuron spiking patterns in a computational model Equivalent stimulation (*I*_stim_ = 60 μA cm^−2^) was applied in all panels. Coloured traces show the relative activation (i.e. $g/\bar{g}$) of the added conductance. *A*, the starting model (with leak conductance, fast‐activating sodium conductance and delayed rectifier potassium conductance) exhibited tonic spiking. *B*, adding a low‐threshold, non‐inactivating (K_v_1‐type) potassium conductance (${\bar{g}}_{K,\mathrm{lt}}$ = 6 mS cm^−2^) yielded single spiking. Inset shows horizontally enlarged view of the shaded region on the main trace to highlight that the initial spike occurs before *g*_K,lt_ activates, and that all subsequent spikes are prohibited since *g*_K,lt_ does not inactivate. *C*, adding an inactivating (A‐type) potassium conductance (${\bar{g}}_{K,A}$ = 8 mS cm^−2^) to the starting model yielded delayed spiking. In this case, spiking occurs only after *g*_K,A_ inactivates, as highlighted in the inset, which again shows a horizontally enlarged view of the shaded region on the main trace. *D*, decreasing ${\bar{g}}_{K,A}$ (to 5 mS cm^−2^) yielded gap spiking by allowing an initial spike to occur before activation of *g*_K,A_ but subsequent spikes are delayed until *g*_K,A_ inactivates. *E*, adding *g*_K,lt_ (= 6 mS cm^−2^) and *g*_K,A_ (= 8 mS cm^−2^) to the starting model yielded reluctant spiking because fast activation of *g*_K,A_ prevents the initial spike while sustained activation of *g*_k,lt_ prevents late spikes even once *g*_K,A_ inactivates. *F*, summary of conductance changes in *A–E*.

Using a simple conductance‐based model, we sought to identify the minimal changes in ion channel expression required to convert the model between spiking patterns. The starting model – which contains only fast‐activating sodium conductance, delayed rectifier potassium conductance, and a leak conductance – spikes tonically (Fig. 2 *A*). The ion channels we added to this model were chosen based on past work by us (Prescott *et al*. 2008; Ratté *et al*. 2015) and others (Grudt & Perl, 2002; Ruscheweyh & Sandkuhler, 2002; Graham *et al*. 2007b, 2008; Smith *et al*. 2015). Adding low‐threshold non‐inactivating (K_v_1‐type) potassium conductance *g*_K,lt_ converted the model to single spiking (Fig. 2 *B*) whereas adding an inactivating (A‐type) potassium conductance *g*_K,A_ converted it to delayed spiking (Fig. 2 *C*). The activation profiles show how each potassium current shapes the spiking pattern. A single spike can occur before *g*_K,lt_ activates, but all subsequent spikes are prevented because *g*_K,lt_ does not inactivate. In contrast, *g*_K,A_ activates quickly enough to prevent spiking at the onset of stimulation, but late spikes occur once *g*_K,A_ inactivates. Gap spiking (Fig. 2 *D*), which resembles a mixture of single and delayed spiking, occurs when ${\bar{g}}_{K,A}$ is low enough that a single spike can occur despite rapid activation of *g*_K,A_, but other spikes are delayed until *g*_K,A_ inactivates. Including high enough densities of *g*_K,A_ *and g*_K,lt_ disallowed single and delayed spiking, thus yielding reluctant spiking (Fig. 2 *E*).

Other ion channels contribute to shaping SDH neuron response properties, such as subthreshold sodium and calcium conductances that encourage temporal summation in tonic‐spiking neurons (Prescott & De Koninck, 2005; Ratté *et al*. 2015) and adaptation conductances that encourage phasic spiking (Prescott & Sejnowski, 2008). But together, *g*_K,lt_ and *g*_K,A_ are sufficient to explain qualitative differences in the pattern of initial spiking, which is the primary basis for electrophysiological classification of SDH neurons. Fig. 2 *F* shows these spiking patterns relative to the 2‐D space defined by the densities of *g*_K,lt_ and *g*_K,A_.

### Quantitative mapping of conductance density to spiking pattern

**Figure 3:**
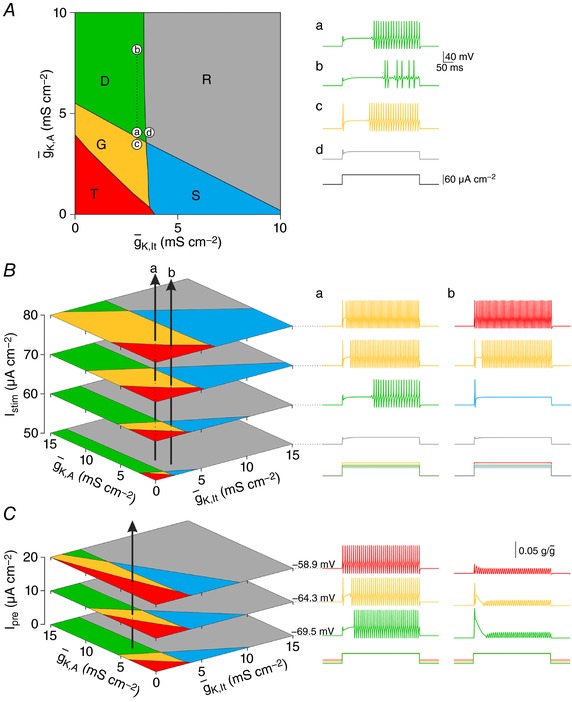
Relationship between ion channel densities and spiking pattern *A*, systematically co‐varying ${\bar{g}}_{K,\mathrm{lt}}$ and ${\bar{g}}_{K,A}$ revealed that distinct regions in this 2‐D parameter space yield different spiking patterns. Boundaries shown here are based on testing with *I*_stim_ *= *60 μA cm^−2^. Traces in *a–d* show sample responses for parameter values labelled on the main plot. Large parameter variations that remain within a region yield the same spiking pattern; compare condition *a* (${\bar{g}}_{K,\mathrm{lt}}$ = 3 mS cm^−2^, ${\bar{g}}_{K,A}$ = 4 mS cm^−2^) with condition *b* (${\bar{g}}_{K,A}$ increased by 4 mS cm^−2^). In contrast, small parameter variations that cross a boundary yield different spiking patterns; compare condition *a* with condition *c* (${\bar{g}}_{K,A}$ reduced by 0.5 mS cm^−2^) or condition *d* (${\bar{g}}_{K,\mathrm{lt}}$ increased by 0.5 mS cm^−2^). *B*, boundaries can shift because of stimulus intensity (*I*_stim_), meaning a neuron with fixed values of ${\bar{g}}_{K,\mathrm{lt}}$ and $\bar{g}$_K,A_ can exhibit different spiking patterns at different *I*_stim_. To illustrate, each vertical arrow on the left panel represents a neuron: for neuron *a*, ${\bar{g}}_{K,\mathrm{lt}}$ = 3 mS cm^−2^ and ${\bar{g}}_{K,A}$ = 4 mS cm^−2^; for neuron *b*, ${\bar{g}}_{K,\mathrm{lt}}$ = 3.5 mS cm^−2^ and ${\bar{g}}_{K,A}$ = 2.5 mS cm^−2^. The spiking pattern at each *I*_stim_ (illustrated on the right) depends on which region the arrow passes through. *C*, boundaries can also shift because of pre‐stimulus membrane potential. For these simulations, a subthreshold ‘pre‐pulse’ (*I*_pre_) was used to vary the membrane potential before the onset of suprathreshold stimulation. Each plane represents the response to *I*_stim_ *= *60 μA cm^−2^ after a different pre‐pulse (pre‐stimulus membrane potential is indicated beside each voltage trace). The vertical arrow represents a neuron with ${\bar{g}}_{K,\mathrm{lt}}$ = 2 mS cm^−2^ and ${\bar{g}}_{K,A}$ = 6 mS cm^−2^. Traces on the right show the reduced availability of *g*_K,A_ depending on *I*_pre_. By partially inactivating *g*_K,A_, subthreshold depolarization reduces the availability of those channels for activation during suprathreshold stimulation, effectively re‐scaling the *y*‐axis.

Next, we systematically co‐varied ${\bar{g}}_{K,\mathrm{lt}}$ and ${\bar{g}}_{K,A}$ to quantify the impact on spiking. The resulting plot (Fig. 3 *A*) shows five regions, each associated with a different spiking pattern. Whereas broadly separated conductance densities within a region yield the same spiking pattern (notwithstanding quantitative differences), narrowly separated conductance densities straddling a boundary yield distinct patterns (compare traces *a–d*). However, those boundaries can shift depending on stimulus intensity (Fig. 3 *B*) or pre‐stimulus membrane potential (Fig. 3 *C*), meaning the same neuron may exhibit different spiking patterns under different test conditions. For Fig. 3 *B* and *C*, each arrow represents a neuron whose spiking pattern (for each test condition) is predicted by the region pierced by that arrow. The sample traces verify these predictions. Traces in the right panel of Fig. 3 *C* show relative activation of *g*_K,A_; less of this conductance is available for activation at the onset of *I*_stim_ when pre‐stimulus membrane potential is depolarized by a pre‐pulse *I*_pre_. These results are consistent with experimental observations illustrated in Fig. 1 *B*–*D*.

**Figure 4:**
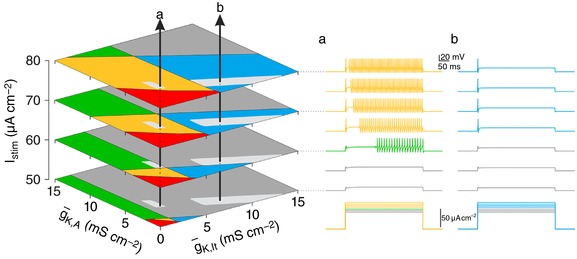
Estimating ion channel densities from spiking pattern sequences Spiking patterns were determined for *I*_stim_ between 50 and 80 μA cm^−2^, at 5 μA cm^−2^ increments for two neurons labelled *a* and *b* on left. Planes are shown for only a subset of *I*_stim_. To estimate the channel densities in neurons *a* and *b*, we determined all combinations of $\bar{g}$_K,A_ and $\bar{g}$_K,lt_ that could produce that sequence. The grey patches shown on each plane together demarcate the volume in which each arrow must exist. The spiking pattern sequence for neuron *a* leads to a more tightly constrained estimate of ion channel densities than does the sequence for neuron *b*.

On the surface, classification is compromised by the sensitivity of spiking patterns to test conditions. Indeed, if a neuron's spiking pattern is classified using only one or two stimulus intensities and without any regard for membrane potential, the classification has little value. On the other hand, if those sensitivities are thoroughly documented, that information can be used to help infer the ion channel densities in that neuron. To illustrate, Fig. 4 shows sample responses from two model neurons, but now, rather than predicting the spiking patterns at each stimulus intensity based on where the arrows intersect each plane (as in Fig. 3), we invert the problem to ask in what volume each arrow must pass to account for the specific sequence of spiking patterns. A simple algorithm (see Methods) was developed to determine all combinations of $\bar{g}$_K,lt_ and $\bar{g}$_K,A_ (shown as light grey regions) that account for the spiking pattern sequences. These areas are smaller than the spiking pattern regions because a sequence of spiking patterns across different stimulus intensities is rarer than the spiking pattern at any one intensity. Likewise, certain spiking pattern sequences are rarer than others: a rare sequence (as for neuron *a*) will give a more tightly constrained prediction of the underlying ion channel densities than a more common sequence (as for neuron *b*). For the same reason, testing more stimulus intensities and/or membrane potentials will help refine the prediction. Inferring ion channel densities in this way works best for neurons that operate near a bifurcation (i.e. whose spiking patterns are sensitive to test conditions).

### Estimating spiking pattern proportions from ion channel density distributions

**Figure 5:**
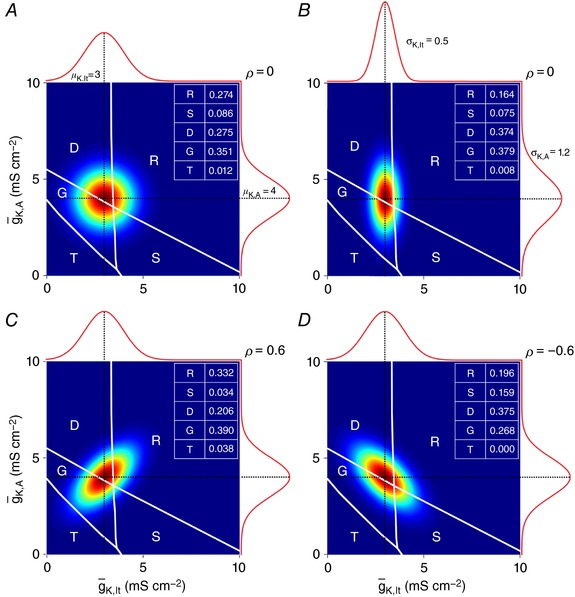
Estimating spiking pattern proportions from ion channel density distributions Within a set of neurons, $\bar{g}$_K,lt_ and $\bar{g}$_K,A_ are likely to exhibit variability consistent with a Gaussian distribution. Univariate distributions describing $\bar{g}$_K,lt_ and $\bar{g}$_K,A_, which are represented by curves on the edges of each graph, combine to give a joint distribution represented by colour (where dark red indicates the highest probability). In all panels, μ_K,lt_ = 3 mS cm^−2^ and μ_,A_ = 4 mS cm^−2^. *A*, if the widths of the two univariate distributions are equal (σ_K,lt_ = σ_K,A_ = 1 mS cm^−2^), the joint probability distribution is circular. *B*, if the widths are unequal (σ_K,lt_ decreased to 0.5 mS cm^−2^ and σ_K,A_ increased to 1.2 mS cm^−2^), the joint probability distribution becomes elliptical. Correlations between and ${\bar{g}}_{K,\mathrm{lt}}$ and ${\bar{g}}_{K,A}$, although not reflected in the univariate distributions, are important for describing the joint distribution, with a positive correlation (ρ > 0) yielding a slanted ellipse whose long axis has a positive slope (*C*), whereas a negative correlation (ρ < 0) yields slanting in the other direction (*D*). Since the total volume under these joint probability distributions equals 1, the volume sitting over each spiking pattern region represents the proportion of neurons exhibiting that pattern. Spiking pattern proportions are shown in the table on each plot.

Whereas each neuron is represented by a single point on the *x–y* plane based on its particular expression of *g*_K,lt_ and *g*_K,A_, a set of neurons of the same type will be represented by a cloud of points. The distribution of those points was assumed to be Gaussian based on random variation in ${\bar{g}}_{K,\mathrm{lt}}$ and ${\bar{g}}_{K,A}$. The univariate distributions describing ${\bar{g}}_{K,\mathrm{lt}}$ and ${\bar{g}}_{K,A}$ are represented by bell‐shaped curves shown respectively on the *x*‐ and *y*‐axes of plots in Fig. 5. These univariate distributions combine to form a joint (or bivariate) distribution represented by colour on each plot. If ${\bar{g}}_{K,\mathrm{lt}}$ and ${\bar{g}}_{K,A}$ are uncorrelated (i.e. expression of one channel is independent of the other channel), the joint distribution will be circular when the standard deviations of the two univariate distributions are equal (Fig. 5 *A*) or elliptical when the standard deviations are unequal (Fig. 5 *B*). If ${\bar{g}}_{K,\mathrm{lt}}$ and ${\bar{g}}_{K,A}$ are positively or negatively correlated (quantified as the correlation coefficient ρ), the joint distribution will take a slanted elliptical shape but, notably, this is not reflected in the univariate distributions (Fig. 5 *C* and *D*).

Within a set of neurons, different neurons may exhibit distinct spiking patterns if the joint distribution of ion channel densities straddles one or more spiking pattern boundaries. Thus, to describe spiking within a set of neurons, one must determine the *proportion* of different spiking patterns (see insets on Fig. 5). We can estimate those proportions by projecting the joint distribution of ion channel densities onto the spiking pattern regions and calculating the portion of that distribution that sits over each region (see Methods). To do this, one must know the mean (μ) and standard deviation (σ) of the univariate distributions describing ${\bar{g}}_{K,\mathrm{lt}}$ and ${\bar{g}}_{K,A}$, plus the correlation coefficient (ρ). Each univariate distribution can be characterized with experiments conducted in separate sets of neurons, but determining ρ requires measurements of ${\bar{g}}_{K,\mathrm{lt}}$ *and* ${\bar{g}}_{K,A}$ in the same neuron, which can be prohibitively difficult (e.g. drugs used to isolate one current for voltage clamp measurements may preclude measurement of the other current). Consequently, correlations are often neglected despite theoretical work showing that they are important (Marder & Taylor, 2011). As shown in Fig. 5, differences in correlation can yield very different spiking pattern proportions.

### Estimating ion channel density distributions from spiking pattern proportions

**Figure 6:**
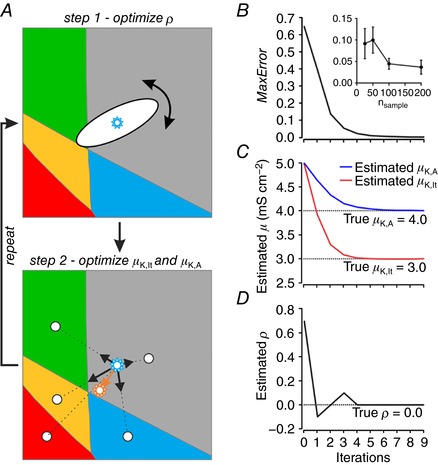
Estimating ion channel density distributions from spiking pattern proportions *A*, schematic representation of the iterative, two‐step process used to estimate the joint distribution of ion channel densities (μ_K,lt_, μ_,K,A_ and ρ) from an observed (target) set of spiking pattern proportions. Starting with arbitrarily chosen parameter values for μ, in step 1, ρ is systematically varied in 0.1 increments to find the value yielding the best match between *estimated* and *target* spiking pattern proportions. That value is carried forward to step 2, in which estimates of μ_K,lt_ and μ_,K,A_ are updated by drawing vectors from the centre of the joint distribution to the centroid of each spiking pattern region. The length of each vector was adjusted based on the error (see Methods) and the distribution is shifted to the position given by the net vector. Step 1 was then repeated with the updated values of μ_K,lt_ and μ_,K,A_, and so on, until the error decreased below a threshold, after which point ρ was systematically varied in 0.01 increments for greater accuracy. The algorithm continued until the MaxError reached a minimum (*B*) and estimates of μ (*C*) and ρ (*D*) stabilized, which occurred within a few iterations. Data in *B–D* show results of fitting the spiking pattern proportions from a target distribution with μ_K,lt_ = 3 mS cm^−2^, μ_,K,A_ = 4 mS cm^−2^, ρ = 0 and σ_K,lt_ = σ_K,A_ = 1 mS cm^−2^. In additional tests, *n* samples were drawn randomly from the target distribution and spiking pattern proportions were determined from the fraction of samples falling within each spiking pattern region. Regardless of how target spiking pattern proportions were generated, the estimation process was the same. Inset in panel *B* shows MaxError at steady‐state (mean ± standard deviation) as a function of *n*_sample_, where each data point is based on five tests.

Given the difficulty of measuring correlations in ion channel expression, we sought to invert the approach used in Fig. 5 (i.e. predicting spiking pattern proportions from the joint distribution of ion channel densities) to instead predict ion channel density distributions, most notably ρ, from spiking pattern proportions. To solve this optimization problem, we developed an algorithm that finds the joint distribution of ion channel densities best able to account for a given proportion of spiking patterns. Estimated values of parameters μ_K,lt_, μ_K,A_, and ρ are optimized through a two‐step process that is repeated iteratively to minimize the error between *target* and *predicted* spiking pattern proportions, as summarized in Fig. 6 *A* (see Methods for details). Using an arbitrary distribution, a target spiking pattern proportion was calculated as in Fig. 5 or a number of samples (*n*_sample_) were drawn randomly from that distribution and the target proportion was calculated from the fraction of samples falling within each spiking pattern region. The latter approach was used to test the effects of finite sampling. Results of fitting are reported for target proportion determined through the former method unless otherwise indicated.

To start the fitting process, an initial distribution with σ_K,lt_ = σ_K,A_ = 1 mS cm^−2^ was created at the centre of the plot (μ_K,lt_ = μ_K,A_ = 10 mS cm^−2^ for a 20 × 20 mS cm^−2^ plane). Neither the starting values of μ nor the dimensions of the plot impact the final outcome (data not shown). The fitting process was repeated for different values of σ (see below). The spiking pattern proportions yielded by this distribution (i.e. the estimated proportions) were calculated as in Fig. 5. In step 1, the value of ρ was systematically varied and predicted proportions were re‐calculated; the value of ρ yielding the smallest error was carried forward to the next step. In step 2, values of μ_K,lt_ and μ_K,A_ were updated by using the error to scale vectors pointing from the centre of the joint distribution to the centroid of each spiking pattern region, the rationale being to pull the distribution towards regions whose spiking pattern was underestimated and push it away from regions whose spiking pattern was overestimated. The updated values of μ_K,lt_ and μ_K,A_ were carried forward to a second iteration of step 1, during which ρ was re‐optimized using updated values of μ. These two fitting steps were repeated until the MaxError was minimized (Fig. 6 *B*) and estimates of μ (Fig. 6 *C*) and ρ (Fig. 6 *D*) stabilized, which typically occurred within a few iterations. The inset in Fig. 6 *B* shows MaxError at steady‐state plotted as a function of the number of neurons used to estimate the target spiking pattern proportion (see above). That relationship argues that reasonably large data sets (∼100 neurons or more) are needed to reliably estimate the target spiking pattern proportion.

**Figure 7:**
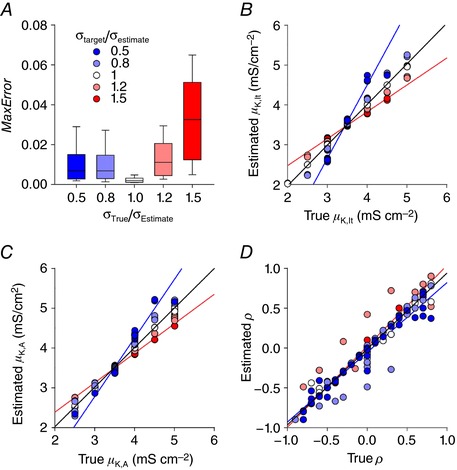
Sensitivity of ion channel density estimation to assumed value of σ Sets of spiking pattern proportions were calculated for joint distributions with arbitrarily chosen parameter values. σ_estimate_ was fixed at 1 mS cm^−2^ but, unlike previously fitted distributions, σ_target_ ( = σ_K,lt_ and σ_K,A_) took a range of values. Specifically, the algorithm was applied to target distributions where σ_estimate_ correctly estimated σ_target_ = 1.0 mS cm^−2^ (σ_target_/σ_estimate_ = 1.0), σ_target_ was 20% greater or less than estimated (σ_target_/σ_estimate_ = 1.2 or 0.8, respectively), or σ_target_ was 50% greater or less than assumed (σ_target_/σ_estimate_ = 1.5 or 0.5, respectively). *N* = 40 target distributions belonging to each group were used for testing, each with arbitrarily chosen values of μ_K,lt_, μ_,K,A_ and ρ. *A*, box‐plot shows the MaxError in the estimated spiking pattern proportions, with box ends and whisker ends representing the 1st/3rd quartiles and 5th/95th percentile, respectively. The median error value of 0.002 when σ was correctly estimated (σ_target_/σ_estimate_ = 1.0) was significantly less than when σ was misestimated (*P* < 0.05, Tukey tests). Estimations of μ_K,lt_ (*B*), μ_,K,A_ (*C*) and ρ (*D*) are shown relative to their true values. Black lines show the regression using all points; their slopes were all within 5% of the expected value of 1. However, when the regression was calculated using points from σ_target_/σ_estimate_ = 0.5 (blue) or 1.5 (red), 5 of the 6 values deviated significantly from the expected value of 1 (*P* > 0.05, one sample *t* tests with Bonferroni correction).

To test our method, we fitted sets of spiking pattern proportions generated using arbitrarily chosen ion channel density distributions. As summarized in Table 1 for the estimation of sample distributions shown in Fig. 5 *A*, *C* and *D*, our algorithm was very successful in estimating the ion channel density distribution based on spiking pattern proportions. However, for those examples, estimated values of σ (denoted σ_estimate_) were set to the true values of σ (denoted σ_true_) rather than being fitted. To explore the effects of misestimating σ, and thus establish if σ must also be fitted, we fixed σ_estimate_ at 1 mS cm^−2^ and generated joint distributions with σ_K,lt_ = σ_K,A_ = [0.5, 0.8, 1.0, 1.2, 1.5] mS cm^−2^. Although all errors were < 5%, they were significantly lower when σ_estimate_ = σ_true_ compared with when σ_estimate_ ≠ σ_true_ (*P* < 0.001, Kruskal–Wallis test; *P* < 0.05 for each pairwise comparison to σ_true_/σ_estimate_ = 1, Tukey tests). Comparing estimated values of μ and ρ against their true values (Fig. 7 *B*–*D*) similarly revealed that those estimates were degraded when σ_estimate_ ≠ σ_true_.

**Table 1:** Testing the algorithm with different target neuron population distributions

	Population A		Population C		Population D	
	Target	Estimated	Target	Estimated	Target	Estimated
Parameter						
μ_K,lt_ (mS cm^−2^)	3	2.998	3	2.999	3	2.997
μ_,K,A_ (mS cm^−2^)	4	4.002	4	4.003	4	4.003
ρ	0.00	0.00	0.60	0.61	−0.60	−0.60
Proportions						
Tonic (T)	0.274	0.273	0.332	0.333	0.196	0.195
Single (S)	0.086	0.086	0.034	0.033	0.159	0.158
Delayed (D)	0.275	0.276	0.206	0.206	0.375	0.376
Gap (G)	0.351	0.351	0.390	0.390	0.268	0.268
Reluctant (R)	0.012	0.012	0.038	0.038	0.000	0.000
MaxError						
From volume	0.001		0.001		0.001	
*n*_sample_ * = *200	0.0368 ± 0.0162		0.0261 ± 0.0142		0.0334 ± 0.0189	
*n*_sample_ * = *100	0.0445 ± 0.0126		0.0461 ± 0.0187		0.0330 ± 0.0133	
*n*_sample_ * = *50	0.0997 ± 0.0303		0.0692 ± 0.0237		0.0911 ± 0.0374	
*n*_sample_ * = *25	0.0915 ± 0.0349		0.1263 ± 0.0765		0.1112 ± 0.0931	

Population *A*, *C*, and *D* refer to conditions shown in the corresponding panels of Fig. 5. MaxError values for target proportions based on random sampling are mean ± standard deviation based on five tests for each condition.

**Figure 8:**
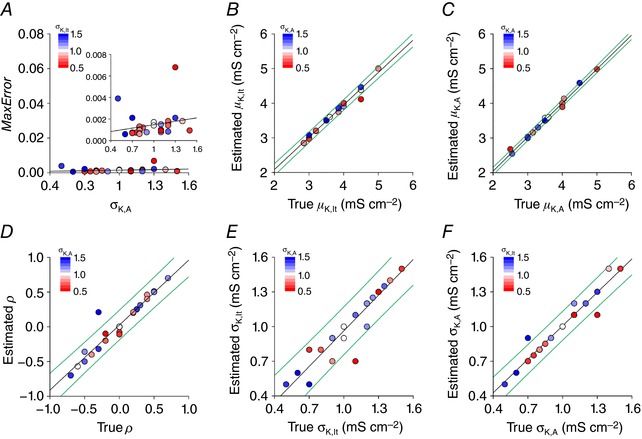
Estimating ion channel distributions, including σ, from spiking pattern proportions Values of σ_estimate_ for σ_K,lt_ and σ_K,A_ were independently varied between 0.4 and 1.7 mS cm^−2^ by increments of 0.1 mS cm^−2^. The two‐step algorithm explained in Fig. 6 was re‐run for all combinations of σ_estimate_ values. Values of σ_K,lt_ and σ_K,A_ yielding the least error after fitting the remaining parameters (μ_K,lt_, μ_,K,A_, ρ) were considered the best estimates. This approach was tested on *N* = 29 target distributions. *A*, MaxError plotted against true σ_K,A_ on the *x*‐axis and σ_K,lt_ represented in colour. Scaling of the *y*‐axis on the main graph is the same as for Fig. 7 *A* for comparison; inset shows enlarged view. Slope of the regression line did not deviate significantly from 0 (*P* > 0.05, one‐sample *t* test). Plotting estimated *vs*. true values of μ_K,lt_ (*B*), μ_,K,A_ (*C*), ρ (*D*), σ_K,lt_ (*E*) and σ_K,A_ (*F*) revealed the accuracy with which each parameter was estimated. Regression lines are shown in black, with the 95% prediction intervals shown in green. No regression line slopes deviated significantly from the expected value of 1 (*P* < 0.05, one sample *t* tests with Bonferroni correction).

Having established the need to fit σ, we tried adding a σ‐fitting step into our algorithm. However, this caused the algorithm to run very slowly. We found that it was more efficient to simply re‐run the algorithm with different σ_estimate_ values and identify *a posteriori* which gave the best fit of the spiking pattern proportions. When this was done, error values fell (compare Fig. 8 *A* to Fig. 7 *A*) and did not systematically differ with σ (*P* > 0.05, one sample *t* test). Moreover, all five parameters were accurately estimated over a range of values (Fig. 8 *B*–*F*); specifically, the slope of all regression lines fell within 7% of (and none differed significantly from) the expected value of 1 (*P* > 0.05, one sample *t* tests with Bonferroni correction). These results demonstrate that values of all μ and σ could be estimated to within approximately ±0.2 mS cm^−2^ and ρ could be estimated within ±0.2 based on the conditions tested. Sources of neuronal heterogeneity not accounted for in our neuron model and any inaccuracies in the initial measurement of spiking pattern proportions will tend to reduce performance below these benchmarks (see Discussion).

## Discussion

In this study, we reproduced five of the spiking patterns observed in SDH neurons by varying the densities of just two ion channels, namely, a low‐threshold non‐inactivating potassium conductance *g*_K,lt_ and an inactivating (A‐type) potassium conductance *g*_K,A_. Systematically co‐varying those two conductances revealed boundaries that represent the transition between spiking patterns. Le Franc and Le Masson (2010) used a similar approach to study spiking patterns in deep dorsal horn neurons, but we are not aware of previous studies like this in the superficial dorsal horn. The boundaries we found imply that subtle changes in ${\bar{g}}_{K,\mathrm{lt}}$ or ${\bar{g}}_{K,A}$ can cause a neuron to switch spiking patterns. Yet the regions in parameter space associated with each spiking pattern are themselves quite large, implying that the same spiking pattern (notwithstanding quantitative differences) can arise from a broad range of ${\bar{g}}_{K,\mathrm{lt}}$ and ${\bar{g}}_{K,A}$, so long as a boundary is not crossed. This combination of observations has important implications for (i) classifying neurons based on spiking pattern and (ii) ascribing different spiking patterns to unique ion channel expression patterns. We will discuss each point and will summarize the tools we have developed to address these issues.

Anyone with first‐hand experience recording from SDH neurons will appreciate that classifying those neurons by spiking pattern is more of an art than a science. The same is true to varying degrees for other neuron populations, but this is not the sort of information that is well documented in publications. That said, some SDH cell types are more distinguishable than others. For example, tonic‐spiking neurons tend to spike repetitively over a broad range of stimulus intensities, irrespective of pre‐stimulus membrane potential, and they also exhibit features such as rebound spiking and a biphasic afterhyperpolarization that distinguishes them from other cell types (Prescott & De Koninck, 2002). Punnakkal *et al*. (2014) found that nearly all genetically defined GABAergic and glycinergic neurons in the SDH were tonic spiking, which contrasts with the heterogeneity they observed for glutamatergic neurons (see below). Although other cell types can spike repetitively, especially in response to strong stimulation, they tend to exhibit single or delayed spiking for stimulus intensities near rheobase. And unlike the voltage‐insensitivity of tonic spiking, single‐spiking neurons can switch to reluctant spiking and delayed‐spiking neurons can switch to gap spiking depending on membrane potential (Fig. 1 *C*). Such observations cast doubt on whether distinctions between single and reluctant spiking or between delayed and gap spiking are legitimate. But more importantly, those observations reveal that some neurons operate near spiking pattern boundaries (or bifurcations). We have shown here that boundaries shift with changes in stimulus intensity or pre‐stimulus membrane potential (Fig. 3), thus allowing a given neuron to exhibit more than one spiking pattern. Indeed, our simulations specifically predict voltage‐dependent switching between single and reluctant spiking, and between delayed and gap spiking. The observation of temperature‐dependent switching between delayed and reluctant spiking (Graham *et al*. 2008) is also consistent with our simulations insofar as those two spiking patterns also share a boundary. Specifically, temperature will quantitatively alter ion channel gating, but would not be expected to qualitatively alter the spiking pattern unless a bifurcation occurred.

An association between single and delayed spiking may seem unlikely given how distinct those patterns are, yet our simulations indicate that these two patterns can arise from similar ion channel densities. Gap spiking helps reveal the ‘missing link’ between them. Notably, single and delayed spiking can occasionally be observed in the same neuron (Fig. 1 *D*) and are disproportionately found in neurons with multipolar (or radial/vertical) morphology (Prescott & De Koninck, 2002) and in neurons defined by expression of calretinin (Smith *et al*. 2015) or vesicular glutamate transporter type 2 (Punnakkal *et al*. 2014). Conversely, neither single nor delayed spiking is commonly observed in inhibitory neurons defined by expression of parvalbumin (Hughes *et al*. 2012) or prion protein (Ganley *et al*. 2015). Of all the spiking patterns, phasic spiking tends to be the most promiscuous. Together, these observations suggest that tonic spiking occurs almost exclusively amongst inhibitory neurons whereas delayed, gap, single and reluctant spiking are associated with excitatory neurons. That said, the four different spiking patterns do not imply that there are four different excitatory cell types (each defined by expression of a distinct set of ion channels); instead, those four spiking patterns likely represent a single cell population in which variations in ${\bar{g}}_{K,\mathrm{lt}}$ and ${\bar{g}}_{K,A}$ straddle the intersection of two spiking pattern boundaries.

The above conclusion is seemingly inconsistent with Abraira *et al*. (2016) who reported delayed spiking in certain sets of inhibitory neurons and tonic spiking in certain sets of excitatory neurons. But the samples of delayed spiking they showed for inhibitory neurons lack the inflection association with an A‐type potassium current (see Fig. 1 *A*) and the sample of tonic spiking they showed for a parvalbumin‐expressing excitatory neuron could be considered phasic spiking (see Fig. 1 *B*). Their ‘regular spiking’ cells align better with our definition of tonic spiking. In other words, the inconsistencies are arguably superficial, based on nuanced criteria that form the basis for this sort of phenomenological classification. The potential for confusion speaks to the need for mechanism‐based classification schemes. In other words, functional classification should become more of a science and less of an art. Cluster analysis based on quantifiable metrics is a must, but the choice of metrics should be guided by putative ionic mechanisms (e.g. measuring the delay to spiking for test stimuli applied at different membrane potentials to rule in/out the contribution of an A current). The modelling and quantitative analysis presented in this study aim to help shift the field in that direction.

Though spiking pattern‐based classification is compromised by the sensitivity of spiking patterns to test conditions, carefully documenting that sensitivity can help constrain estimates of the underlying ion channel densities (Fig. 4). Moving from the characterization of single neurons to the characterization of neuronal populations, spiking pattern heterogeneity can help inform our understanding of the underlying distributions of ion channel density. As demonstrated in Figs 6, 7, 8, the proportion of different spiking patterns observed within a population can be used to estimate ion channel density distributions, including any correlations in the expression of different ion channels. We are not aware of any past studies that have used this approach and, in that respect, the success of our algorithm represents proof‐of‐principle demonstration that this approach can work. But notably, such an approach requires that data are collected in a standardized way, and from many neurons (to ensure that the population is appropriately sampled). Even then, our modelling has neglected sources of neuronal heterogeneity that may compromise the ability to estimate ion channel distributions. First, even if our model included all the ion channels expressed in real neurons, the densities of channels that we did not systematically vary (i.e. leak conductance, fast sodium conductance, and delayed rectifier potassium conductance) would also vary between neurons, and this additional heterogeneity would blur the spiking pattern boundaries plotted on planes defined by ${\bar{g}}_{K,\mathrm{lt}}$ and ${\bar{g}}_{K,A}$. That said, >2 ion channel densities could be co‐varied during initial mapping, in which case boundaries currently depicted as lines on a plane would become manifolds within a higher dimensional space; this is harder to visualize but is still computationally feasible. Second, real neurons express other ion channels that were not included in our model, and even if those channels are not necessary to explain each spiking pattern, heterogeneity in their expression may also contribute to blurring the spiking pattern boundaries. Addressing these and other sources of heterogeneity (e.g. expression of ion channels in the dendrites and variations in dendritic morphology) is computationally feasible but requires larger data sets than can be acquired by painstakingly patching one neuron at a time. As higher throughput recording methods are developed, and larger data sets are collected, analysis of those data using these computational tools will become practicable. The collation of data across labs into databases like NeuroElectro (Tripathy *et al*. 2014) will also facilitate such efforts. But even if the data are available, one must develop a valid computational model of the cells of interest in order to fit its parameters to the data.

Our results also highlight how correlations in ion channel expression may influence spiking pattern proportions. Specifically, in Fig. 5 we show that the same univariate distributions of ${\bar{g}}_{K,\mathrm{lt}}$ and ${\bar{g}}_{K,A}$ can yield different spiking pattern proportions depending on if and how the densities of those channels are correlated. Such correlations are often overlooked, but can reveal themselves during computational modelling, when the average neuron (in functional terms) is not recapitulated by endowing the model with the average densities of conductances known to be expressed in that cell type (Golowasch *et al*. 2002). Correlations in ion channel expression levels have been documented experimentally (MacLean *et al*. 2003; Bergquist *et al*. 2010; Cao & Oertel, 2011), as have correlations in the voltage dependencies of co‐expressed channels (Amendola *et al*. 2012). Such correlations emerge via co‐regulation of ion channels by homeostatic (O'Leary *et al*. 2013) or neuromodulatory (Khorkova & Golowasch, 2007) processes, and are important for enabling robust regulation of cellular function (Zhao & Golowasch, 2012; Golowasch, 2014). But whereas most past work has focused on maintaining cellular function on the basis of different ion channel combinations, where compensation for one channel by another channel naturally leads to correlations (Hudson & Prinz, 2010), our results suggest that functional heterogeneity may also depend on correlations. The algorithm we have developed provides a novel tool to predict correlations on the basis of that heterogeneity.

To conclude, we have used computational modelling to reproduce five of the spiking patterns by which SDH neurons are often classified. Our results demonstrate that different combinations of two potassium channels can account for that heterogeneity. By mapping the relationship between channel densities and spiking pattern, our results reveal the relationship between different spiking patterns (i.e. which patterns share a boundary), which in turn predicts the switching between certain spiking patterns based on factors like stimulus intensity or membrane potential. Together with past observations, our data suggest that certain spiking patterns do not reflect cell types with distinct ion channel expression patterns, but, rather, suggest that certain spiking patterns result from continuous variations in ion channel densities that manifest distinct spiking patterns because of the inherent non‐linearity of the spike generation process. These are important issues to consider for making neuron classification schemes more robust and for ensuring efficient identification of the molecular basis of important functional differences.

## Additional information

### Competing interests

The authors have declared that no competing interests exist

### Author contributions

Simulations were performed in the laboratory of SAP. AB and SAP conceived the design of the study. AB conducted all simulations. AB and SAP analysed and interpreted the data. AB and SAP wrote the manuscript. SAP supervised the project. Both authors have read and approved the final version of the manuscript, and agree to be accountable for all aspects of the work. All persons designated as authors qualify for authorship, and all persons qualified for authorship are listed as authors.

### Funding

This work was supported by a Discovery Grant from the Natural Sciences and Engineering Research Council (NSERC) of Canada and by an Ontario Early Researcher Award to SAP.
